# Characteristics of Hospitals Eligible for Rural Emergency Hospital Designation

**DOI:** 10.1001/jamahealthforum.2022.4613

**Published:** 2022-12-09

**Authors:** Paula Chatterjee, Matthew J. Klebanoff, Qian Huang, Amol S. Navathe

**Affiliations:** 1 Department of Medicine, Perelman School of Medicine at the University of Pennsylvania, Philadelphia

## Abstract

This cross-sectional study compares the characteristics, finances, services, and challenges at hospitals that are eligible vs not eligible to become rural emergency hospitals.

## Introduction

The Consolidated Appropriations Act of 2021 introduced the rural emergency hospital (REH) designation to preserve emergency and outpatient services in areas unable to sustain full-service hospitals.^1,2^ An REH must maintain a 24-hour emergency department but will not provide inpatient care. Hospitals that convert into REHs starting in 2023 will receive a 5% add-on to Medicare outpatient prospective payment rates and a new facility payment. This program represents a fundamental change in rural health care delivery. Although previous work has identified hospitals likely to convert into REHs,^3^ little is known about the characteristics, finances, and services of eligible hospitals.

## Methods

The Perelman School of Medicine Institutional Review Board deemed this cross-sectional study exempt from review. We followed the STROBE reporting guideline.

We used the 2019 Medicare Cost Reports to distinguish REH-eligible hospitals, defined as critical access or rural hospitals with 50 or fewer beds, from noneligible rural hospitals. Rurality was ascertained using non–metropolitan statistical areas. We obtained hospital staffing and services data from the 2019 American Hospital Association (AHA) Annual Survey. Hospital finances were winsorized at 5th and 95th percentiles and summarized with means using the 2016 to 2019 Medicare Cost Reports. We ascertained county-level availability of hospital beds and physicians from the 2020 to 2021 Area Health Resources File. Because the REH program seeks to promote equity in rural communities,^2,4^ we obtained county-level population demographics, including self-reported race and ethnicity, from the 2021 County Health Rankings and 2010 zip code–level rural-urban commuting area codes of the Department of Agriculture (eMethods and eTable in the Supplement).

We compared characteristics, finances, and services between eligible and noneligible hospitals. We focused on services the program seeks to promote: emergency, outpatient, and telehealth.

We used unpaired, 2-tailed *t* tests and Wilcoxon tests to compare continuous variables and χ^2^ tests for categorical variables. After adjusting for multiple comparisons, the significance threshold was 2-sided *P* < .01 for characteristics and finances and *P* < .02 for services. Analyses were performed with SAS, version 9.4 (SAS Institute).

## Results

There were 1569 REH-eligible and 368 noneligible rural hospitals (Table 1). Eligible hospitals were located in counties with fewer hospital beds per 100 square miles compared with noneligible hospitals (4.0 vs 12.4; *P* < .001). Eligible hospitals had more emergency physicians per 1000 patient-days (0.16 vs 0.07; *P* < .001), but fewer county-level primary care physicians per 100 000 population (55.8 vs 62.1; *P* < .001), and were located in counties with a lower percentage of Black population (5.9% vs 11.1%; *P* < .001) compared with noneligible hospitals.

**Table 1.** Characteristics of Hospitals Eligible for REH Designation and Noneligible Rural Hospitals

Characteristic	Mean (SD)		Difference (95% CI)^a^	*P* value
	Eligible hospitals for REH designation (n = 1569)	Noneligible rural hospitals (n = 368)		
Hospital beds, No. (%)	25.4 (9.9)	117.0 (65.6)	−91.3 (−98.1 to −84.6)	<.001
Medical-surgical beds per 100 square miles, county-level^b^	4.0 (12.5)	12.4 (45.4)	−8.4 (−13.1 to −3.7)	<.001
Ownership, No. (%)				
Nonprofit	838 (53.4)	224 (60.9)	−7.5 (−13.0 to −1.9)	.01
For-profit	109 (6.9)	78 (21.2)	−14.2 (−18.6 to −9.9)	<.001
Government	622 (39.6)	66 (17.9)	21.7 (17.1 to 26.3)	<.001
Teaching status, No. (%)	28 (1.8)	82 (22.3)	−20.5 (−24.8 to −16.2)	<.001
Critical access status, No. (%)	1204 (76.7)	0	76.7 (74.6 to 78.8)	<.001
Metropolitan area, No. (%)	140 (8.9)	12 (3.3)	5.7 (3.4 to 8.0)	<.001
Micropolitan area, No. (%)	310 (19.8)	293 (79.6)	−59.9 (−64.4 to −55.3)	<.001
Small town, No. (%)	694 (44.2)	56 (15.2)	29 (24.6 to 33.4)	<.001
Rural area, No. (%)	425 (27.1)	7 (1.9)	25.2 (22.6 to 27.8)	<.001
Case-mix index	1.2 (0.2)	1.5 (0.2)	−0.2 (−0.3 to −0.2)	<.001
Health system member, No. (%)	745 (47.5)	246 (66.8)	−19.4 (−24.8 to −14.0)	<.001
Staffing, total per 1000 patient-days				
Hospital employees	20.7 (61.5)	16.5 (34.9)	4.2 (−0.5 to 8.9)	.08
Employed physicians	1.32 (4.30)	0.85 (2.28)	0.5 (0.1 to 0.9)	.02
Hospitalist physicians	0.09 (0.42)	0.08 (0.12)	0	.45
Emergency physicians	0.16 (0.51)	0.07 (0.20)	0.1 (0.1 to 0.1)	<.001
PCPs per 100 000 population, county-level^b^	55.8 (32.1)	62.1 (25.3)	−6.3 (−9.3 to −3.2)	<.001
Finances				
Operating margin, %^c^	−1.0 (8.7)	3.5 (8.0)	−4.5 (−5.4 to −3.5)	<.001
Total margin, %^d^	1.9 (7.2)	4.9 (7.6)	−3 (−3.8 to −2.1)	<.001
Outpatient PPS payments				
In millions, $	4.7 (3.8)	14.7 (11.8)	−10 (−11.2 to −8.8)	<.001
As % of total Medicare payments^e^	65.9 (15.6)	41.6 (9.4)	24.3 (23.1 to 25.6)	<.001
Inpatient PPS payments				
In millions, $	2.5 (2.6)	19.9 (18.1)	−17.4 (−19.2 to −15.5)	<.001
As % of total Medicare payments^e^	32.8 (13.6)	56.3 (9.9)	−23.5 (−24.7 to −22.3)	<.001
Uncompensated care, %^f^	8.0 (4.4)	7.2 (3.1)	0.8 (0.4 to 1.2)	.13
Medicaid share, %^g^	11.0 (10.3)	21.9 (7.6)	−11 (−11.9 to −10.1)	<.001
Occupancy, %	31.7 (17.5)	42.4 (15.6)	−10.7 (−12.5 to −8.9)	<.001
Race and ethnicity and socioeconomic demographic, county-level^b^				
County population	54 889 (318 969)	5452 (28 309)	362 (−15 705 to 16 429)	.96
% American Indian or Alaska Native	3.5 (10.1)	2.5 (8.5)	1 (0.0 to 2.0)	.05
% Asian	1.1 (2.6)	1.2 (2.3)	−0.1 (−0.3 to 0.2)	.66
% Hispanic	9.4 (13.4)	7.3 (11.0)	2.1 (0.8 to 3.4)	.002
% Non-Hispanic Black	5.9 (12.2)	11.1 (16.2)	−5.2 (−7.0 to −3.4)	<.001
% Non-Hispanic White	78.7 (19.6)	76.4 (20.1)	2.4 (0.1 to 4.7)	.04
Unemployment rate	4.5 (1.7)	5.0 (1.4)	−0.4 (−0.6 to −0.2)	<.001
Median household income per y, $	49 163 (10 325)	46 279 (9194)	2884 (1813 to 3956)	<.001
Uninsured rate in adults, %	13.2 (6.1)	13.2 (5.9)	−0.1 (−0.8 to 0.6)	.82
High school completion rate, %	87.9 (8.0)	87.3 (6.8)	0.6 (−0.2 to 1.4)	.13

Abbreviations: PCP, primary care physician; PPS, prospective payment system; REH, rural emergency hospital.

^a^ Difference between eligible and noneligible hospitals show absolute differences for variables reported as means and percentage point differences for all other variables.

^b^ County-level variables reflect the county in which the hospital is located. All other variables were estimated at the level of the hospital.

^c^ Operating margin reflects a narrow measure of hospital profitability based on patient care–related revenue.

^d^ Total margin reflects a broader measure of overall hospital profitability.

^e^ Total Medicare payments for critical access hospitals were calculated as follows: payments received under the critical access hospital program – sequestration adjustment for critical access hospitals + Medicare outpatient payments, lesser of costs or charges. Total Medicare payments for non–critical access hospitals were calculated as follows: inpatient prospective payments + outpatient prospective payments.

^f^ Uncompensated care indicates the sum of charity care and bed debt as a fraction of operating expenses.

^g^ Medicaid share indicates the share of Medicaid inpatient days out of total inpatient days.

Eligible hospitals had lower operating (−1.0% vs 3.5%; *P* < .001) and total margins (1.9% vs 4.9%; *P* < .001) than noneligible hospitals. Outpatient payments composed a larger share of total Medicare payments at eligible vs noneligible hospitals (65.9% vs 41.6%; *P* < .001). Eligible hospitals were less likely than noneligible hospitals to provide certain emergency services, including orthopedic (52.7% vs 92.2%; *P* < .001) and obstetric care (41.0% vs 94.0%; *P* < .001) (Table 2), outpatient psychiatric (21.3% vs 29.5%; *P* < .001) and substance use disorder care (4.1% vs 16.0%; *P* < .001), and several telehealth services.

**Table 2.** Services Available at Hospitals Eligible for REH Designation and Noneligible Rural Hospitals^a^

Service	Hospitals, No. (%)		Percentage point difference (95% CI)	*P* value
	Eligible hospitals for REH designation (n = 1103)	Noneligible rural hospitals (n = 281)		
Emergency services^b^				
ED	1092 (98.1)	279 (99.3)	−1.2 (−2.4 to 0.1)	.17
Adult cardiology	295 (26.5)	222 (79.0)	−52.5 (−57.9 to −47.1)	<.001
Diagnostic catheterization	33 (3.0)	172 (61.2)	−58.2 (−64.0 to −52.5)	<.001
Orthopedics	586 (52.7)	259 (92.2)	−39.5 (−43.8 to −35.2)	<.001
Neurology	150 (13.5)	162 (57.7)	−44.2 (−50.3 to −38.1)	<.001
Obstetrics care	456 (41.0)	264 (94.0)	−53 (−57.0 to −49.0)	<.001
Outpatient and ambulance services				
Hospital-based outpatient care^c^	869 (78.1)	235 (83.6)	−5.6 (−10.5 to −0.6)	.04
Primary care department^d^	626 (56.2)	142 (50.5)	5.7 (−0.8 to 12.2)	.09
Outpatient surgery	938 (84.3)	278 (98.9)	−14.7 (−17.1 to −12.2)	<.001
Outpatient psychiatric care	237 (21.3)	83 (29.5)	−8.2 (−14.1 to −2.4)	.003
Outpatient SUD care	46 (4.1)	45 (16.0)	−11.9 (−16.3 to −7.4)	<.001
Ambulance^e^	579 (52.0)	141 (50.2)	1.8 (−4.7 to 8.4)	.58
Telehealth services^e^				
Consultation	631 (56.7)	161 (57.3)	−0.6 (−7.1 to 5.9)	.86
ICU	144 (12.9)	72 (25.6)	−12.7 (−18.2 to −7.2)	<.001
Stroke	445 (40.0)	186 (66.2)	−26.2 (−32.4 to −20.0)	<.001
Postdischarge monitoring	129 (11.6)	61 (21.7)	−10.1 (−15.3 to −4.9)	<.001
Chronic care monitoring	251 (22.6)	80 (28.5)	−5.9 (−11.7 to −0.1)	.04
Psychiatric or addiction	316 (28.4)	106 (37.7)	−9.3 (−15.6 to −3.1)	.002

Abbreviations: ED, emergency department; ICU, intensive care unit; REH, rural emergency hospital; SUD, substance use disorder.

^a^ Among eligible hospitals, there were 466 (29.7%) with missing service data on all services variables except ED in 2019; among noneligible hospitals, there were 87 (23.6%) with missing data. The bivariate comparison was nonsignificant at a threshold adjusted for multiple comparisons (6.1 percentage points; 95% CI, 1.2-11.0; *P* = .02).

^b^ Emergency services were chosen based on evaluation and treatment required for common, emergency conditions.

^c^ Hospital-based outpatient care refers to any health care services provided within the hospital by appointment on an ambulatory basis.

^d^ Primary care department refers to outpatient primary care provided through a dedicated unit or clinic within the hospital.

^e^ Ambulance and all telehealth services were assigned according to their availability at the hospital, joint venture facility, or health system. Availability of all other services was identified at the hospital level.

## Discussion

Eligible hospitals had poorer baseline finances and provided fewer emergency, outpatient, and telehealth services than noneligible hospitals. These results suggest that facility payments associated with REH conversion, estimated to be $3 million annually per hospital, would be larger than the mean outpatient payment add-on.^5^ These funds, combined with lower operating costs from inpatient care cessation, may be sufficient to sustain emergency and outpatient care. However, whether these resources can counter broader rural health challenges related to workforce shortages and fewer economic opportunities remains unknown.^6^

Study limitations include differential missing data for service variables from the AHA survey and use of prepandemic data on hospital services and finances. Nonetheless, this study offered insights into the financial and service-related challenges in preserving access to rural emergency and outpatient services.

## Notes

Supplement. **eMethods.** Details on Data Sources and Associated References **eTable.** Data Sources and Variables
eReferences
