# Supplementary material for: Characteristics of Hospitals Eligible for Rural Emergency Hospital Designation
Source: JAMA Health Forum. 2022 Dec 9;3(12):e224613. doi: 10.1001/jamahealthforum.2022.4613 (PMC9856250; doi:10.1001/jamahealthforum.2022.4613)
Supplement: Supplement. — eMethods. Details on Data Sources and Associated References eTable. Data Sources and Variables eReferences [file jamahealthforum-e224613-s001.pdf]

Supplementary Online Content

Chatterjee P, Klebanoff MJ, Huang Q, Navathe AS. Characteristics of hospitals eligible for rural emergency hospital designation. *JAMA Health Forum.* 2022;3(12):e224613. doi:10.1001/jamahealthforum.2022.4613

eMethods. Details on Data Sources and Associated References

eTable. Data Sources and Variables

eReferences

This supplementary material has been provided by the authors to give readers additional information about their work.

© 2022 Chatterjee P et al. *JAMA Health Forum.*

eMethods. Details on Data Sources and Associated References

We used the following data sources in this study:

1) Medicare Cost Reports (hospital-level):[1] These data, compiled by the RAND Corporation, comprise a hospital-year dataset of characteristics and financial variables. Hospitals that receive payments from Medicare are required to submit an annual cost report. RAND transforms fiscal year data into calendar year formatting, which were used for this analysis. We limited the sample to general, acute care hospitals. Additional information on this data source is available here: https://www.hospitaldatasets.org/

2) American Hospital Association's Annual Survey (AHA, hospital-level):[2] This is an annual survey of hospital characteristics, staffing levels, and clinical services provided by a hospital. It is voluntary but the substantial majority of general, acute care hospitals respond to this survey each year. Additional information on this data source is available here: https://www.ahadata.com/system/files/media/file/2022/05/2020-AHA-Annual.pdf

3) Area Health Resources File (AHRF, county-level):[3] This is compiled resource that amasses hospital-, county- and national data from a variety of different sources on health care utilization, staffing, and demographics. Individual sources of data that are aggregated into the AHRF include, but are not limited to, the American Medical Association's Master File, Centers for Medicare and Medicaid Services' National Provider Identification file, Census Bureau's Population Estimates Program, and Census Bureau's Small Area Income and Poverty Estimates. Given their aggregation of different sources, different variables in the AHRF may reflect different years of data depending on the primary source. Additional information is available here: https://data.hrsa.gov/Content/Documents/topics/AHRF%20Definition.pdf

4) County Health Rankings (CHR, county-level):[4] This is also a compiled resource that amasses county-level data from different sources to summarize population demographics, health-related behaviors, and socioeconomic indicators. Individual sources of data include, but are not limited to, National Center for Health Statistics (Mortality and Natality files), Behavioral Risk Factor Surveillance System, American Community Survey (5-year estimates), and Bureau of Labor Statistics. Similar to the AHRF, different variables in the CHR may reflect different years of data depending on the primary source. Additional information is available here: https://www.countyhealthrankings.org/2022-measures

5) US Department of Agriculture (USDA, zip code-level):[5] The Economic Research Service at the USDA provides rural-urban commuting area codes, which serve as a granular definition of zip-code level rurality that is based on population density, commuting patterns, and adjacency to urban areas. Additional information is available here: https://www.ers.usda.gov/data-products/rural-urban-commuting-area-codes/documentation/

© 2022 Chatterjee P et al. *JAMA Health Forum.*

eTable. Data Sources and Variables

Data source	Year(s)	Variable	Variable for Linkage	Notes
Medicare Cost Reports	2019	Beds	CMS Certification Number	
	2019	Ownership (non-profit, for-profit, government)	CMS Certification Number	
	2019	Teaching	CMS Certification Number	
	2019	Critical access	CMS Certification Number	
	2019	Case mix index	CMS Certification Number	
	2019	Health system member	CMS Certification Number	
	2016-2019	Operating margin	CMS Certification Number	Winsorized averages at the 5th & 95th percentiles
	2016-2019	Total margin	CMS Certification Number	Winsorized averages at the 5th & 95th percentiles
	2016-2019	Outpatient PPS payments	CMS Certification Number	Winsorized averages at the 5th & 95th percentiles
	2016-2019	Inpatient PPS payments	CMS Certification Number	Winsorized averages at the 5th & 95th percentiles
	2016-2019	Total net patient revenue	CMS Certification Number	Winsorized averages at the 5th & 95th percentiles
	2016-2019	Uncompensated care	CMS Certification Number	Winsorized averages at the 5th & 95th percentiles
	2016-2019	Medicaid share	CMS Certification Number	Winsorized averages at the 5th & 95th percentiles
	2016-2019	Occupancy	CMS Certification Number	Un-winsorized average
American Hospital Association's Annual Survey	2019	Adjusted patient days	CMS Certification Number	
	2019	Total hospital employees	CMS Certification Number	
	2019	Total employed physicians	CMS Certification Number	
	2019	Total hospitalist physicians	CMS Certification Number	
	2019	Total emergency physicians	CMS Certification Number	
	2019	Emergency Department	CMS Certification Number	Binary indicator for whether a hospital provides these services

© 2022 Chatterjee P et al. *JAMA Health Forum.*

	2019	Adult Cardiology	CMS Certification Number	Binary indicator for whether a hospital provides these services
	2019	Diagnostic Catheterization	CMS Certification Number	Binary indicator for whether a hospital provides these services
	2019	Orthopedics	CMS Certification Number	Binary indicator for whether a hospital provides these services
	2019	Neurology	CMS Certification Number	Binary indicator for whether a hospital provides these services
	2019	Obstetrics Care	CMS Certification Number	Binary indicator for whether a hospital provides these services
	2019	Hospital-based Outpatient Care	CMS Certification Number	Binary indicator for whether a hospital provides these services
	2019	Primary Care Department	CMS Certification Number	Binary indicator for whether a hospital provides these services
	2019	Outpatient Surgery	CMS Certification Number	Binary indicator for whether a hospital provides these services
	2019	Psychiatric Outpatient Services	CMS Certification Number	Binary indicator for whether a hospital provides these services
	2019	Substance Use Disorder Outpatient Services	CMS Certification Number	Binary indicator for whether a hospital provides these services
	2019	Ambulance Services	CMS Certification Number	Binary indicator for whether a hospital, joint venture, or health system provides these services
	2019	Telehealth Consultation Services	CMS Certification Number	Binary indicator for whether a hospital, joint venture, or health system provides these services
	2019	Telehealth ICU Services	CMS Certification Number	Binary indicator for whether a hospital, joint

© 2022 Chatterjee P et al. *JAMA Health Forum.*

				venture, or health system provides these services
	2019	Telehealth Stroke Services	CMS Certification Number	Binary indicator for whether a hospital, joint venture, or health system provides these services
	2019	Telehealth Post-Discharge Monitoring	CMS Certification Number	Binary indicator for whether a hospital, joint venture, or health system provides these services
	2019	Telehealth Chronic Care Monitoring	CMS Certification Number	Binary indicator for whether a hospital, joint venture, or health system provides these services
	2019	Telehealth Psychiatric/Addiction Services	CMS Certification Number	Binary indicator for whether a hospital, joint venture, or health system provides these services
Area Health Resources File	2020-2021	County area in square miles	County FIPS code	2020-2021 data from AHRF reflect 2010 Census Redistricting data for this variable.
	2020-2021	County population	County FIPS code	2020-2021 data from AHRF reflect 2020 Census County Pop Estimates for this variable.
	2020-2021	Medical/surgical beds per county	County FIPS code	2020-2021 data from AHRF reflect 2019 AHA Survey data for this variable. Medical/surgical beds per 100 square miles was calculated using the Area Health Resources File, which sums the total number of hospital beds in a county using the American Hospital Association's Annual Survey, and also includes a variable for county size

© 2022 Chatterjee P et al. *JAMA Health Forum.*

				in square miles. The variable represents their quotient, multiplied by 100.
	2017	Primary care physicians per county	County FIPS code	2017 data from AHRF use the American Medical Association's Master File to determine the number of primary care physicians under age 75 within a county. Primary care physicians include M.D.'s and D.O.'s who practice in general family medicine, general practice, general internal medicine, and general pediatrics. Subspecialties within these domains are excluded. Denominators of county population are derived from the US Census and not age-adjusted.
County Health Rankings	2021	Percent American Indian/Alaska Native	County FIPS code	2021 CHR data reflect 2019 Census Population Estimates for this variable
	2021	Percent Asian	County FIPS code	2021 CHR data reflect 2019 Census Population Estimates for this variable
	2021	Percent Hispanic	County FIPS code	2021 CHR data reflect 2019 Census Population Estimates for this variable
	2021	Percent non-Hispanic Black	County FIPS code	2021 CHR data reflect 2019 Census Population Estimates for this variable
	2021	Percent non-Hispanic White	County FIPS code	2021 CHR data reflect 2019 Census Population Estimates for this variable

© 2022 Chatterjee P et al. *JAMA Health Forum.*

	2021	Unemployment Rate	County FIPS code	2021 CHR data reflect 2019 BLS data for this variable
	2021	Median Household Income	County FIPS code	2021 CHR data reflect 2019 Small Area Income and Poverty Estimate data for this variable
	2021	Uninsured Adults Rate	County FIPS code	2021 CHR data reflect 2018 Small Area Health Insurance Estimates for this variable
	2021	High School Completion Rate	County FIPS code	2021 CHR data reflect 5-year estimates from ACS 2015-2019 for this variable
US Department of Agriculture	2010	Rural-urban commuting area codes	Zip code	Codes of 1-3 correspond to metropolitan areas; 4-6 correspond to micropolitan areas; 7-9 correspond to small towns; 10 corresponds to rural areas

CMS=Centers for Medicare and Medicaid Services; PPS=prospective payment system; ICU=intensive care unit; AHRF=Area Health Resources File; AHA=American Hospital Association's Annual Survey; CHR=County Health Rankings; BLS=Bureau of Labor Statistics; ACS=American Community Survey.

© 2022 Chatterjee P et al. *JAMA Health Forum.*

eReferences

1. RAND Hospital Data. RAND Corporation. Accessed October 11, 2022. https://www.hospitaldatasets.org

2. AHA Annual Survey Database. American Hospital Association. Accessed October 11, 2022. https://www.ahadata.com/aha-annual-survey-database

3. Area Health Resources Files. Accessed October 11, 2022. https://data.hrsa.gov/data/download

4. County Health Rankings. Robert Wood Johnson Foundation. Accessed October 11, 2022. https://www.countyhealthrankings.org

5. USDA ERS - Rural-Urban Commuting Area Codes. Accessed October 11, 2022. https://www.ers.usda.gov/data-products/rural-urban-commuting-area-codes/

© 2022 Chatterjee P et al. *JAMA Health Forum.*
